# Impact of Protein Stability, Cellular Localization, and Abundance on Proteomic Detection of Tumor-Derived Proteins in Plasma

**DOI:** 10.1371/journal.pone.0023090

**Published:** 2011-07-29

**Authors:** Qiaojun Fang, Kian Kani, Vitor M. Faca, Wenxuan Zhang, Qing Zhang, Anjali Jain, Sam Hanash, David B. Agus, Martin W. McIntosh, Parag Mallick

**Affiliations:** 1 Fred Hutchinson Cancer Research Center, Seattle, Washington, United States of America; 2 University of Southern California, Los Angeles, California, United States of America; 3 Stanford University, Stanford, California, United States of America; 4 Cedars-Sinai Medical Center, Los Angeles, California, United States of America; University of South Florida College of Medicine, United States of America

## Abstract

Tumor-derived, circulating proteins are potentially useful as biomarkers for detection of cancer, for monitoring of disease progression, regression and recurrence, and for assessment of therapeutic response. Here we interrogated how a protein's stability, cellular localization, and abundance affect its observability in blood by mass-spectrometry-based proteomics techniques. We performed proteomic profiling on tumors and plasma from two different xenograft mouse models. A statistical analysis of this data revealed protein properties indicative of the detection level in plasma. Though 20% of the proteins identified in plasma were tumor-derived, only 5% of the proteins observed in the tumor tissue were found in plasma. Both intracellular and extracellular tumor proteins were observed in plasma; however, after normalizing for tumor abundance, extracellular proteins were seven times more likely to be detected. Although proteins that were more abundant in the tumor were also more likely to be observed in plasma, the relationship was nonlinear: Doubling the spectral count increased detection rate by only 50%. Many secreted proteins, even those with relatively low spectral count, were observed in plasma, but few low abundance intracellular proteins were observed. Proteins predicted to be stable by dipeptide composition were significantly more likely to be identified in plasma than less stable proteins. The number of tryptic peptides in a protein was not significantly related to the chance of a protein being observed in plasma. Quantitative comparison of large versus small tumors revealed that the abundance of proteins in plasma as measured by spectral count was associated with the tumor size, but the relationship was not one-to-one; a 3-fold decrease in tumor size resulted in a 16-fold decrease in protein abundance in plasma. This study provides quantitative support for a tumor-derived marker prioritization strategy that favors secreted and stable proteins over all but the most abundant intracellular proteins.

## Introduction

Blood-based protein biomarkers indicative of the presence, progression, and phenotype of a tumor are of significant clinical interest for diagnostics and prognostics [1], [2], [3], [4]. One common approach to the discovery of such protein biomarkers is to compare cancer tissues with control materials [5] and select candidates from a list of proteins that are more abundantly expressed in the cancer tissues; any selected candidate must be then subsequently verified in serum or plasma. As there may be dozens or hundreds of differentially abundant proteins identified in such experiments [2] researchers must prioritize potential candidates. In principle one should select those tumor proteins that are most likely to find their way into peripheral blood at detectable levels. Logical arguments regarding the need to give higher priority to abundant tumor proteins, stable proteins, or secreted or extracellular proteins are commonly made [1], [6], [7], [8], yet the absolute or relative values of these attributes have not been quantified. In general, the attributes that allow cellular proteins to find their way into the plasma in detectable levels are poorly understood. In this study we sought to estimate the relative importance of each of these three factors in predicting which proteins derived from a tumor are observed in plasma and which are not.

Ordinarily establishing what portion of a protein's plasma concentration originated in a tumor is challenging as plasma abundance is affected by a combination of factors other than tumor leakage, including endogenous host production. To precisely measure tumor derived proteins in plasma we exploited a mouse xenograft model where tumors are derived from human cancer cell lines. Seventy-three percent of human tryptic peptides are not contained in the murine tryptic peptide database. By restricting our analysis to those peptide sequences that were uniquely human, we were able to distinguish tumor from host proteins. We next annotated the proteins for cellular location, protein stability, number of tryptic peptides, and spectral count in tissue (a measure of relative protein abundance [9], and determined the probability of the protein being identified in plasma. The most readily observed proteins were extracellular and stable ones. Although our primary goal was to determine the properties of tissue proteins that are correlated with detection in plasma, we also compared samples from mice with large and small tumors in order to evaluate the effect of tumor size on the likelihood of observation of particular proteins. Tumor size was strongly, but non-linearly, related to protein abundance in plasma.

The characteristics we evaluated here represent only a small number of factors that relate to the chance that a protein will make a high quality biomarker for any given disease. Factors such as tumor shape, vascularization, nutrient penetration, histology, and location may impact detection of tumor-derived proteins in plasma, and relative abundance and variation of a protein in cancer-free plasma will also impact the utilization. Although this model may not completely reflect what can be expected in human disease, or even in other murine models, due to differences in tumor burden and localization, the characteristics that determine the likelihood of detecting a tumor protein in plasma should be transferable to human systems and will serve as a guide for biomarker prioritization.

## Results

### Protein identification from tumor and plasma samples

In order to identify tumor-derived proteins detectable in blood samples by mass-spectrometry-based proteomics, mice carrying tumors of human cell origin were generated. A431 cells sensitive (A431s) or resistant (A431gr derived in vivo as described in Materials and Methods) to gefitinib were mixed with Matrigel and subcutaneously injected into the flanks of nude athymic BALB/c female mice. One group of animals with each tumor type (5 mice) was treated with gefitinib and the other was not (vehicle control). For the A431s model, the average size of tumors in untreated mice was 2500 mm^3^, three times the volume of tumors from treated mice (750 mm^3^) approximately 17 days post implant. In mice with A431gr tumors, the tumor sizes in treated and untreated mice were the same, around 1300 mm^3^ after 18 days of implantation.

Tumor tissue and plasma were harvested on day 17 or 18 post injection. Pooled tumor and plasma samples from treated mice were labeled with heavy acrylamide and samples from untreated animals were labeled with light acrylamide as previously described [10]. Equal masses of protein from the treated and untreated mice were mixed. As a result, four samples were generated, each was a mixture of either tumor or plasma samples from either gefitinib treated or untreated mice. Tumor and plasma samples were analyzed using mass-spectrometry-based proteomics techniques. In order to minimize false positive detection of murine peptide sequences, we employed a stringent set of peptide and protein filters, using only peptides having PeptideProphet [11] probability greater than 0.95 and requiring each protein group to have at least 2 unique peptides meeting that criteria (see Materials and Methods). A total of 2,506 human proteins were identified, including 2,487 from the tumor analysis and 138 from plasma (Table 1). One hundred nineteen (119) proteins were observed in both tumor and plasma. Thus, approximately 5% of tumor proteins were detected in plasma. A total of 445 and 395 mouse-specific proteins were identified in the A431s and A431gr experiments, respectively. This indicates that human-specific tumor proteins make up approximately 20% of all proteins identified in plasma based on our strict filtering criteria. Less than 10% of the proteins characterized in plasma were ambiguously of the mouse or human origin; these proteins were not included in subsequent analyses. Protein identifications were subject to Ontology analysis. Overall, about 90% of the proteins identified had a cellular location annotation by GO. Of the tumor proteins observed in plasma, 40% originated from the extracellular space. In contrast, only 10% of all proteins identified in tumor tissue were extracellular. Thus, there was a four-fold enrichment of extracellular protein identifications in plasma compared to all identified tumor proteins. A list of these proteins is provided in Supplementary Table S1.

**Table 1:** Summary of human tumor proteins identified and quantified in each experiment and their cellular locations.

Xenograft mouse model	Average size of tumors	Tissue type	Proteins identified	Proteins quantified	Cellular location		
					Extracellular	Non-extracellular	Not annotated
**A431s**	Treated: 2500 mm^3^Untreated: 750 mm^3^	plasma	103	18	42	54	7
		tumor	2314	1153	170	1882	262
**A431gr**	Treated = untreated = 1300 mm^3^	plasma	87	18	38	42	7
		tumor	2099	979	163	1705	231

### Tumor protein levels depend on tumor burden

**Figure 1:**
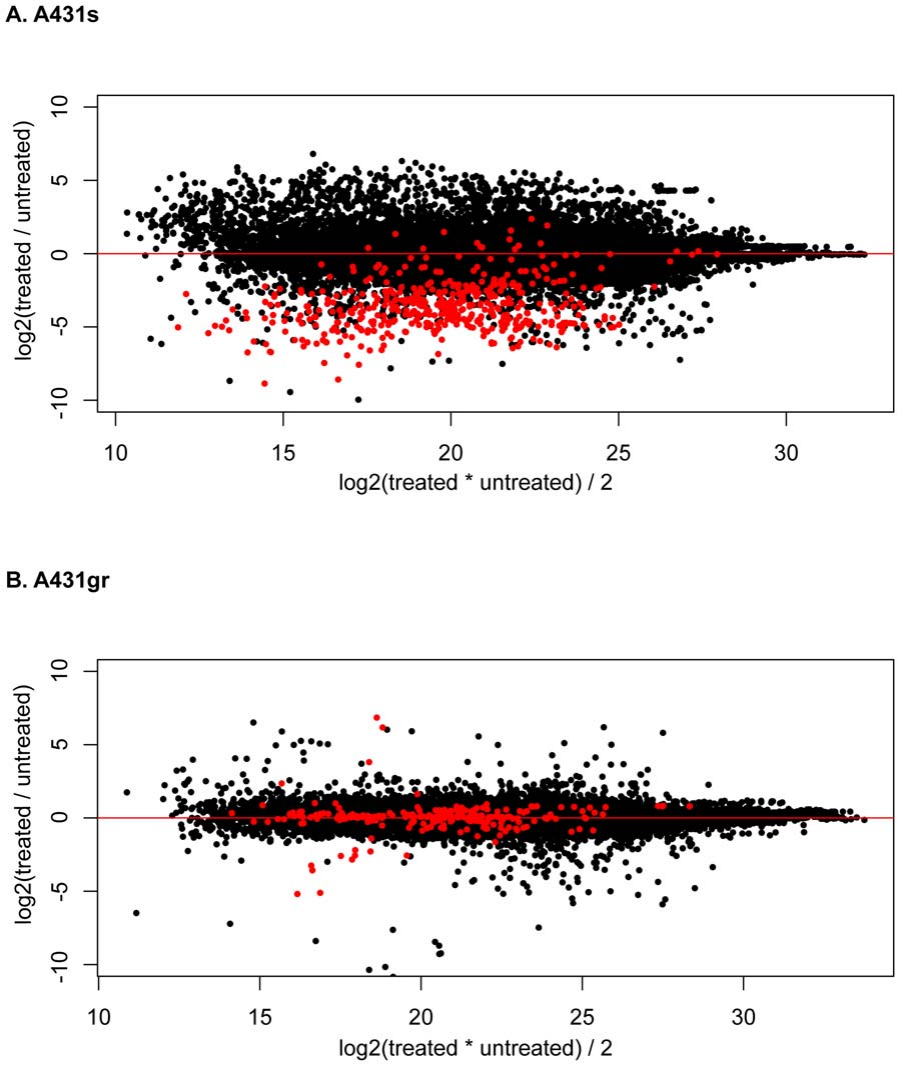
MA plots of (A) A431s and (B) A431gr tumor and mouse proteins in plasma. X axis is the average intensity of MS peaks in treated and untreated samples and Y axis represents the log2(treated/untreated) ratios. Treated samples were labeled with C13 acrylamide and untreated samples were labeled with C12 acrylamide as described in methods. Red points are tumor proteins and black points are mouse proteins.

**Figure 2:**
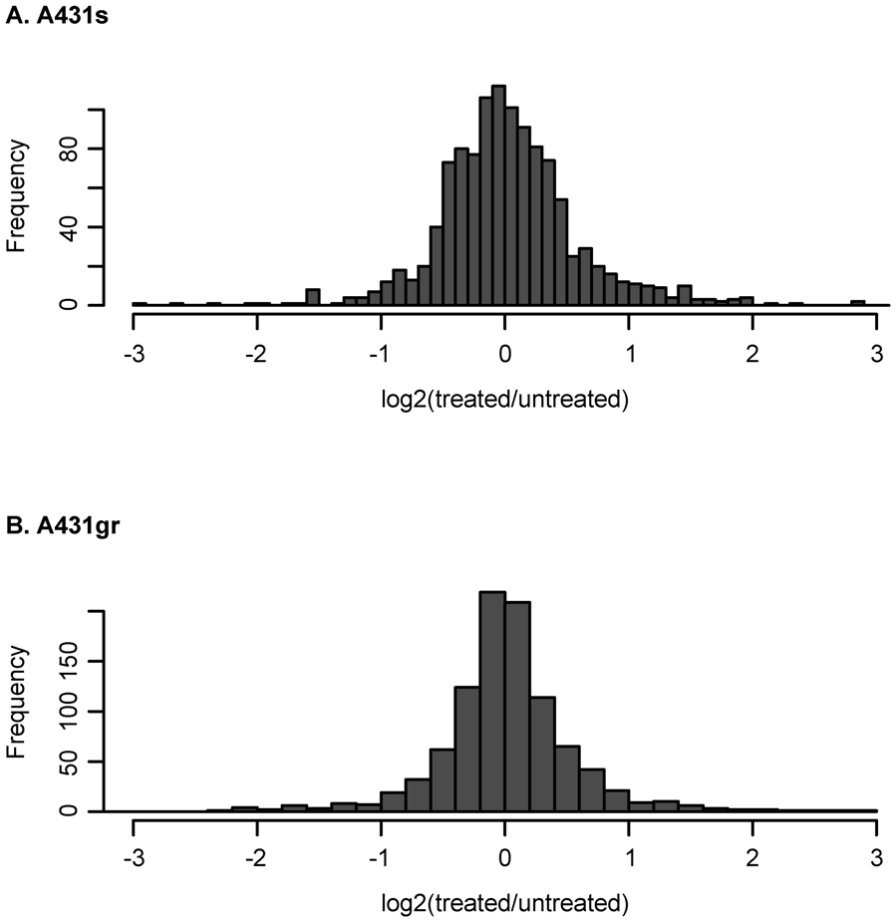
Histograms of (A) A431s and (B) A431gr tumor protein treated/untreated ratios. Ratios are in log2 scale.

All plasma samples contained mixtures of plasma of treated and untreated mice. In the resistant tumor the treated and untreated tumors were the same size, but the sensitive tumor was one-third the size of the untreated. This provides an opportunity to investigate the relationship of tumor burden on the chance that a protein is observed. MA plots in Figure 1 shows the distribution of log2 (treated/untreated) ratios by ion intensity, revealing that the relative tumor protein abundance in plasma from A431s-tumor bearing mice treated with gefitinib was 16 fold (log2(treated/untreated) ratios of -4) lower than those from untreated mice (red points in Figure 1A). However, mouse proteins (black points in Figure 1A) showed no systematic changes in treated vs. untreated samples, thus ruling out variation in loading volume or other specimen processing artifacts as the cause of this difference. This as well suggests that gefitinib treatment does not grossly change the protein expression in host. We also compared the same log2(treated/untreated) ratios of both tumor and mouse plasma proteins from A431gr-tumor bearing mice treated and untreated with gefitinib, which have the same size of tumors and found no systematic changes (Figure 1B). Thus it is reasonable to assume that the consistent difference of tumor proteins in the plasma of A431s-tumor bearing mice with and without treatment is primarily resulted from differences in tumor size in treated and untreated animals. As the ratio of tumor volume in A431s/A431gr animals was 1∶3, it is likely that factors in addition to tumor size must convey a substantial influence on tumor protein levels in murine plasma in order to result in a 1∶16 factor in plasma. Several potential factors could explain the non-linear relationship of the tumor size and abundance in plasma. One could be the change of protein constituents due to drug treatment, which cannot be ruled out by the analyses presented above. However, when we compared the treated and untreated A431s and A431gr tumors of all proteins identified, we found that log2(treated/untreated) ratios of both tumors are normally distributed around 0 (Figure 2), which means on a proteome-wide scale there is no systematic difference of protein abundance between treated and untreated tumors (Figure 2). Moreover, protein level changes in plasma are not associated with changes in tumor; that is, proteins that do not decrease in tumor due to treatment had the same average decrease in plasma. Another potential could be that treatment changes the dynamics of protein secretion, or has an impact on the host ability to remove proteins from circulation. Together this observation suggests that the dynamics of protein production, secretion, and degradation/elimination is not well understood and deserved further attention.

### Extracellular, stable and abundant tumor proteins are more readily detected in plasma

**Figure 3:**
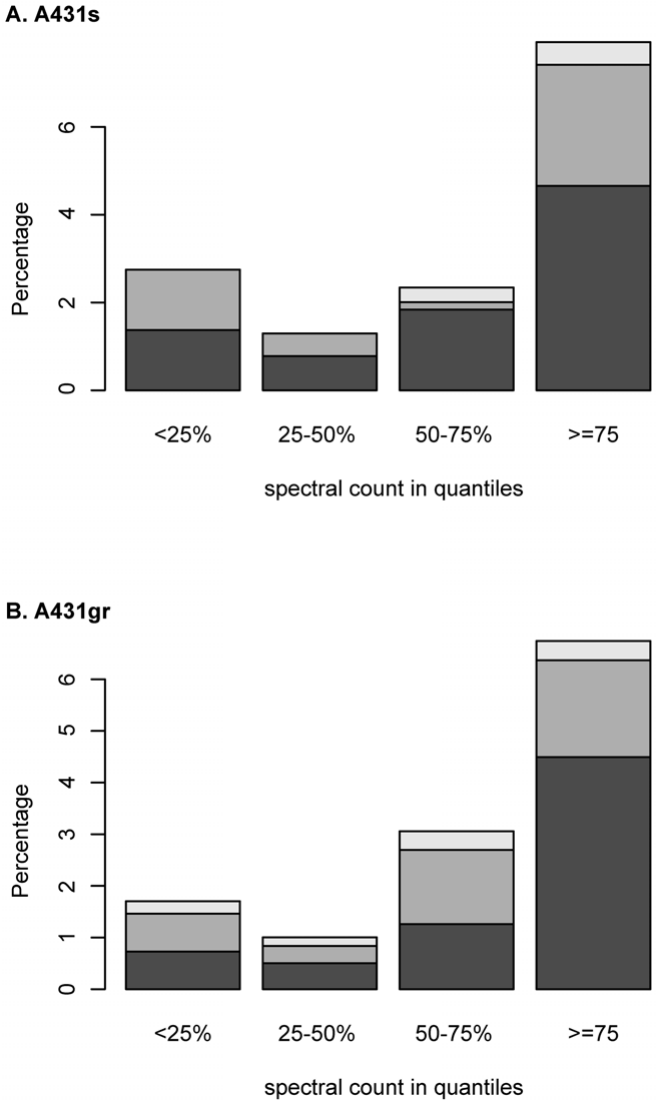
Percentage of (A) A431s and (B) A431gr tumor proteins observed in plasma shown by spectral count in quantile scale and different cellular locations. X axis is spectral counts plotted in quartile scales and increase from left to right. Y axis is the percentage of all tumor proteins identified in plasma. Black bars: non-extracellular; grey bars: extracellular proteins; empty bars: not annotated. Spectral counts were plotted in quartile scale and increase from left to right.

Protein cellular location was annotated by Gene Ontology database as described in Materials and Methods. While 8.7% of all proteins were identified in solid tumors are extracellular, 44% of the tumor proteins detected in plasma are extracellular proteins - a five-fold enrichment. We then evaluated the association between protein stability and the likelihood of detection. Protein stability was estimated from the amino acid sequence using a commonly used method (in the ExPASy proteomics tools) generated by Guruprasad et al. [12], which is based on the correlation of protein stability and its dipeptide composition. The stability of a protein can be represented by a protein instability index score by averaging the dipeptide instability weight values derived from statistically analysis of unstable and stable proteins Guruprasad et al. [12]. Here, we divided protein instability index scores into four quantiles from low to high and count the number of proteins in each category. We found that as instability index scores increased (low protein stability), the likelihood of detection in plasma decreased (Table 2). However, there was no correlation between stability and probability of detection in tumor tissue. The difference of these two patterns was statistically significant as evaluated by the chi-square test (*p*<0.001). Next, we examined how protein abundance influenced the detection of tumor proteins in plasma. We re-coded the spectral counts observed into quantiles representing low, medium-low, medium-high, and high abundance proteins. Percentages of proteins observed in plasma are plotted according to their respective quantiles in spectral counts and color coded to represent different cellular locations in Figure 3. More than 8% of tumor proteins with spectral counts greater than 75% quantile were observed, but less than 3% of proteins identified in plasma fall in each of the lower spectral counts quantiles (Figure 3A and 3B). This demonstrates that highly abundant proteins (quantile > = 75%) were identified more readily in plasma than lowest abundance proteins. Plasma proteins with low spectral counts (quantile >25%) showed an equal ratio of extracellular proteins to non-extracellular proteins. However, proteins with high spectral counts (quantile > = 75%) were enriched for non-extracellular proteins. This suggests that the probability that a non-extracellular protein will be present in plasma is more dependent on the protein abundance than that of extracellular proteins.

**Table 2:** Numbers of human tumor proteins observed or not observed in plasma by instability index.

	Observed in plasma				Not observed in plasma				Chi-square test
instability index	< = 25%	25-50%	50-75%	>75%	< = 25%	25-50%	50-75%	>75%	
**A431s**	37	20	12	11	542	558	566	568	7.3e-05
**A431gr**	32	22	12	11	493	503	512	514	0.001

### Prediction of protein cellular location, stability, and abundance association with the probability of observation of tumor proteins in plasma

We used a logistic regression model to estimate how the cellular location, stability, and abundance associate with the presence of tumor proteins in plasma. This method revealed the impact of each parameter on probability of protein detection. The coefficients and significance (p values) are listed in Table 3; the *p* values and magnitudes from individual analyses and the joint analysis for all of the respective features are shown. For a low probability event, the increase in probability is close to the exponential value of the regression coefficients. Our data indicate that cellular location had the largest single impact on the probability that a protein will be detected in the plasma: Extracellular proteins were 7 (e^1.95^∼7) times more likely to be observed in plasma than non-extracellular proteins, assuming all other factors are the same. Protein stability and abundance also influenced the likelihood of protein detection. Stable proteins (those with instability scores of less than 40, see Materials and Methods) were about twice as likely to be detected in plasma as less stable proteins (e^0.87^∼2.4 times for A431s data and e^0.51^∼1.7 times for A431gr data). In addition, the probability of detection of a tumor protein was higher by 50% (e^0.44^∼1.55) if its spectral count was doubled, all else similar. The number of tryptic peptides was not significantly associated with the likelihood of detection; this was surprising given that proteins that have more tryptic peptides have more chance of being sampled by MS/MS. Neither coefficients nor *p* values changed significantly when the logistic regression was performed in the marginal mode rather the multivariate mode.

**Table 3:** Association of cellular location, protein stability, abundance, and number of tryptic peptides of human tumor proteins with presence in plasma using logistic regression.

		multivariate		marginal	
		Coefficient	P value	Coefficient	P value
**A431s**	Extracellular	1.95	7.1e-13	1.91	1.2e-13
	Stability	0.87	0.001	1.06	9.8e-06
	Spectral counts	0.44	9.4e-13	0.43	3.8e-15
	# of tryptic peptides	-0.008	0.05	-0.0008	0.82
**A431gr**	Extracellular	2.01	9.9e-12	2.00	8.2e-13
	Stability	0.51	0.076	0.81	0.002
	Spectral counts	0.45	3.8e-12	0.41	3.9e-13
	# of tryptic peptides	-0.007	0.098	0.001	0.74

**Figure 4:**
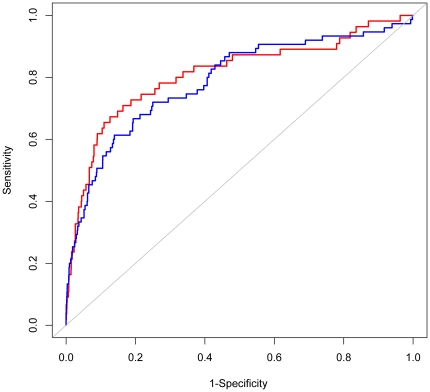
Receiver operating Characteristic (ROC) curves showing the prediction of tumor proteins detected in plasma by using A431s data to predict proteins in A431gr xenograft model (red) or using A431gr data to predict proteins in A431s xenograft model (blue).

To validate the use of a logistic regression model in predicting factors that affect tumor proteins to be detected in plasma, receiver operating characteristic (ROC) curve was used. ROC curves were plotted for our classification model, using one data set (A431s/A431gr) as training set to predict which proteins would be observed in plasma for the other (A431gr/A431s) [13], area under curves (AUCs) (Figure 4) of both curves were close to 0.8, suggesting that these tests were valid. Selection of 10% of all tumor proteins using the model would capture 60% of all plasma proteins.

## Discussion

Plasma, rather than tissues or other bodily fluids, is the most common source for biomarker discovery [14]. However, only a small fraction of proteins detected in blood are cancer related because most cancer-specific proteins from the tumor tissue are diluted as they leak into the interstitial or proximal fluid and lymph, and are diluted further when they enter the blood [15]. During the course of biomarker exploration using high-throughput, large scale studies such as mass-spectrometry based proteomic techniques, knowledge of which tumor-specific proteins are likely to be observed in plasma will be important for data interpretation and pathway analyses. One approach to identifying plasma biomarkers is to identify proteins that are over-abundant or unique to cancer tissues. Because development of blood-based assays can be time-consuming and costly, often only a subset is then selected for further validation.

We analyzed LC-MS/MS data of tumor tissue and plasma from mice xenografted with human A431 cells that were either sensitive or resistant to gefitinib. Because of sequence heterogeneity, this model allowed us to readily distinguish tumor proteins, which were of human origin, from the host mouse proteins. We found that 20% of the mouse plasma proteins identified are from the tumor, higher than the mass of the tumor to the whole mouse which ranges from 4–10%. However, based on spectral count, the total spectral count of tumor proteins is only 4.5% of all plasma proteins. And it should be noted that certain abundant mouse proteins have been removed prior to the mass spectrometry analysis. The high presence of protein IDs from tumor may result from the abnormal active states of cancer cells. Our analysis revealed that the probability of detection of a tumor protein in plasma was associated with the extracellular location of the protein, higher stability and abundance, and the larger tumor size. Of these factors, cellular location contributed most, i.e. extracellular proteins, even those of low abundance, were often observed in plasma. In contrast, only highly abundant nuclear proteins were observed in plasma. Our findings suggest that researchers should have a strong preference for proteins that are secreted, stable, and avoid proteins that are intracellular unless they are highly abundant. As shown in Figure 4, we used the weights on each of these factors to compute a linear classifier score capable of capturing 60% of the detectable tumor proteins while selecting only 10% of other proteins identified in tissues. The exact weights may differ based on tumor occurring site and other factors, but the relative magnitude of these factors may provide a means for researchers to select a small set of markers from among all possible candidates. The instability index scores of proteins identified formed a normal distribution with a mean value of approximately 40 (Supplementary Figure S1), the cutoff chosen to distinguish the stable and unstable proteins. The distribution of tumor proteins in plasma was left shifted (green), compared to those not detected in plasma (black). This is consistent to the result shown above (Table 2). When we standardized the score and implemented logistic regression, the coefficient became 0.48 with a *p* value of 0.001. This suggests that proteins with scores below 1 standard deviation from the mean (about 16% of the total proteins) were 62% more likely to be detected in the plasma than were less stable proteins.

Tumor protein abundance in plasma was lower in samples from mice with smaller tumors than in those with larger tumors (gefitinib-treated vs. untreated mice with sensitive tumors); however, the ratio of tumor volumes in treated vs. untreated mice was smaller than the ratio of protein abundances. We suspect that this discrepancy is due to the sensitivity of the mass spectrometer. Or it may be that proteins derived from larger tumors with higher stability may be non-linearly enriched or that necrosis within the tissue is volume related. An experiment with plasma and tissues collected from animals with a range of tumor sizes would allow testing of these hypotheses. In this analysis, tumor size difference was result from drug treatment, which may raise the concern of altering protein expression. Our analysis found that although that a small number of protein abundances did vary, drug treatment did not grossly alter protein expression levels of both tumor (see Results and Figure 2) and its host. An alternative way to acquire different sizes of tumor without drug treatment is to collect tumors at different time, which has other uncertain factors such as different mouse ages, too. Proteins varied from the drug treatment warrant further investigation but are not within the scope of this study.

There are many other potential considerations for prioritizing cancer biomarkers, such as proteins that are abundant and endogenous in ordinary human plasma will not be good candidates. The guideline we provided in this analysis is what tumor proteins are most likely to be detected in plasma. Obviously, not all tumor proteins detected in plasma can be satisfying candidates since some of them may have endogenous counterparts. In our list of tumor proteins detected in plasma, 64 out of 138 have endogenous mouse homologues and for some proteins, the mouse homologues are highly abundant. These abundant proteins will not be good markers; the low abundant ones require further analysis and validation of significance of changes in the human plasma under disease condition. In summary, it is important to interrogate as many necessary factors for the selection of a good candidate. Our study suggests that stable proteins excreted by tumor cells should be given highest priority in further studies.

## Materials and Methods

### *In vivo* xenografts and tumor and plasma samples

Matrigel (BD Biosciences, Sparks, MD) was mixed 1∶1 with 2×10^9^ A431 cells (ATCC, Manassas, VA) and subcutaneously injected into the flanks of nude athymic BALB/c female mice obtained from Charles River Breeding Laboratories (Wilmington, MA). The A431 gefitinib-resistant tumor cells were selected by serial passage of A431 subcutaneous xenografts in presence of 50 mg/kg of gefitinib (AstraZeneca, London, UK) for nine months. Animals were maintained in pressurized ventilated cages at the Cedars-Sinai Medical Center Vivarium. All animal experiments were performed as per the institutional guidelines and were approved by the Institutional Animal Care and Use Committee at Cedars-Sinai Medical Center (CSMC) (IACUC Number 001276). Gefitinib was administered orally daily to twenty animals at around 10–12 weeks old. Control animals received vehicle alone (20 animals). The tumor volumes were measured twice a week with a digital vernier caliper and were calculated as: π/6 x (larger diameter) × (smaller diameter). Tumor tissue and plasma were harvested after 17 or 18 days post injection At this time, for the A431s model, the average size of tumors in untreated mice was 2500 mm^3^, three times the volume of tumors from treated mice (750 mm^3^). In mice with A431gr tumors, the tumor sizes in treated and untreated mice were the same, around 1300 mm^3^. The mass ratio of tumor to whole mouse ranges from 4–12%. Frozen tumor pieces from 5 mice were individually ground in liquid nitrogen with the aid of a ceramic mortar and equal masses of individual tumor homogenate were pooled and suspended in RAF buffer. The homogenate was centrifuged for 5 min at 200× g. The supernatant was sonicated on ice for 2 minutes and centrifuged for 1 hr at 12,000× g. The supernatant (soluble fraction) was cleared through a 0.22-µm filter. Sera from 5 mice were pooled and depleted using two MARS-3 columns (Agilent, Santa Clara, CA) connected in tandem with HPLC. The unbound fraction was concentrated to a final concentration of 2 mg/ml. Tumor and plasma samples from animals untreated vs. treated with gefitnib were labeled with C12 vs. C13 acrylamide respectively as described in Faca et al. [10].

### Fractionation of A431 tumor and mouse plasma samples

Tumor samples were fractionated by reversed-phase chromatography using 1 mg of total protein. All samples were reduced with DTT (0.6 mg DTT/mg protein) and alkylated with iodoacetamide (3 mg IA/mg protein) prior to chromatography as described elsewhere [16]. Separation was performed in a POROS R1/10 column (4.6×50 mm, Applied Biosystems, Foster City, CA) at a flow rate of 2.7 ml/min using a linear gradient of 10 to 80% of organic solvent over 30 minutes. The aqueous solvent was 5% acetonitrile/95% water/0.1% trifluoracetic acid; the organic solvent was 75% acetonitrile/15% isopropanol/10% water/0.095% trifluoracetic acid. Fractions were collected at a rate of 3 fractions/minute and 72 fractions were collected. Each fraction was individually digested in solution with trypsin (400 ng/fraction) [17]. Adjacent fractions were combined based on protein chromatography features, resulting in a total of 25 fractions for mass spectrometry analysis. Plasma samples were subjected to two-dimensional fractionation based on previously described Intact-protein Analysis System (IPAS) approach [17], [18], [19], [20]. Basically, the sample was diluted to 10 ml with 20 mM Tris (pH 8.5) in 6% isopropanol, 4 M urea and immediately injected on an anion exchange, Mono-Q 10/100 column (Amersham Biosciences, Piscataway, NJ, USA) for the first dimension of the protein fractionation. The buffer system consisted of solvent A (20 mM Tris, pH 8.5, in 6% isopropanol, 4 M urea pH 8.5) and solvent B (20 mM Tris in 6% isopropanol, 4 M urea, 1 M NaCl). The separation was performed at a flow rate of 4.0 ml/min in a gradient of 0–35% solvent B in 44 minutes; 35–50% solvent B in 3 minutes; 50–100% solvent B in 5 minutes; and 100% solvent B for an additional 5 minutes. A total of 12 pools were collected. Each pool was then subjected to a second dimension of separation by reversed-phase chromatography. The reversed-phase fractionation was carried out on a Poros R2 column (4.6×50 mm; Applied Biosystems) using trifluoroacetic acid/acetonitrile as a buffer system (solvent A, 95% H2O, 5% acetonitrile, 0.1% trifluoroacetic acid; solvent B, 90% acetonitrile, 10% H2O, 0.1% trifluoroacetic acid) at a flow rate of 2.7 ml/min. The gradient was 5% solvent A until the absorbance reached baseline (desalting step) and then 5–50% solvent B in 18 minutes; 50–80% solvent B in 7 minutes; and 80–95% solvent B in 2 minutes. During the run, 72 900-µl fractions were collected. Each fraction was individually digested in solution with trypsin (400 ng/fraction) and the fractions were grouped into 8 pools based on chromatographic features, corresponding to a total of 96 fractions for analysis from each experiment.

### Protein identification and quantification by LC-MS/MS

Protein identification by LC-MS/MS was performed as described previously [17]. Briefly, pools of fractions were individually analyzed by LC-MS/MS in a LTQ-FTICR or LTQ-ORBITRAP mass spectrometer (Thermo-Finnigan, Waltham, MA) coupled to a nanoflow chromatography system (Eksigent, Dublin, CA) using a 25-cm column (Picofrit 75 µm ID, New Objectives, Woburn, MA) packed in-house with MagicC18 resin (Michrome Bioresources, Auburn, CA) over a 90-minute linear gradient. Acquired data was automatically processed using default parameters, except where noted, using the Computational Proteomics Analysis System (CPAS), V8.2 [21]. The tandem mass spectra were searched against version 3.44 of the human IPI (71,884 protein entries) and mouse (version 3.44 with 55,078 protein entries) databases. The searches were performed with X!Tandem (2008.02.01). The mass tolerance for precursor ions was set to 1.5 Daltons. The mass tolerance for fragment ions was set to 0.5 Daltons. A fixed modification of 71.0371 mass units was added to cysteine residues for database searching to account for the acrylamide modification and 3.01 Daltons were used as variable modification to account for the heavy cysteine isotope. All identifications with a PeptideProphet [11] probability greater than 0.95 were submitted to ProteinProphet [22] and each of the subsequent protein identifications were required to have at least two unique peptides with tryptic fragments (1 missed cleavage) with allowance for variable modifications on E  =  −18.011, K = 6.020, M = 15.995, and Q  =  −17.027. In order to assign a species, we required at least one unique human peptide for identification as a human protein, referred to throughout this manuscript as “tumor proteins”. One unique mouse peptide was required for identification as a “mouse protein”. The Q3 algorithm [10], developed to accommodate a three-Dalton mass shift in heavy and light peptides, was used to compute the ratios between the light and heavy isotopic pairs (i.e., the untreated vs. treated changes). A spectral count method [9] was used to estimate the relative abundance of proteins. More specifically, peptide spectra with PeptideProphet [11]probability of greater than 0.95 or an error rate of 1% were counted for each IPI entry identified.

### Comparison of human and mouse database entries

Protein sequences in human database (human IPI v.3.44) and mouse database (mouse IPI v. 3.44) were computationally digested with trypsin and a minimum of seven residues was used to match the X! Tandem default search parameter of the minimum parent fragment. A total of 673,735 human peptides were found and 490,809 (73%) peptides were uniquely in the human database; 182,926 (27%) peptides were also observed in the mouse database.

### Data processing and integration

To facilitate comparisons of protein groups among samples, data were aligned by tracking all proteins that were members of a single ProteinProphet group as described by Fang et al. [23]. This provided an analytic data set with one row for each protein group and specifically a column with values indicating the spectral count for proteins in each sample consistent with that group. The cellular location for each protein sequence was determined using the March 2008 generic GO slim from the GO consortium (http://www.geneontology.org/GO.slims.shtml). GO slim files are reduced ontologies with significantly fewer categories than the complete GO ontology. For example, there were about 2400 distinct cellular component categories in the full ontology as of March 2008. The generic March 2008 GO slim file has only 37 categories, several of which are not present in mammalian cells. The script “map2slim” (available from GO) was used to assign proteins to their nearest GO category and to identify those that are located in the extracellular or plasma membrane. Based on GO definition, the term “extracellular” in this study refers to space outside the plasma membrane and is intended to annotate gene products that are not tightly attached to the cell surface. Therefore, proteins annotated with “extracellular” are basically proteins “secreted” into the medium. In cases that an IPI had multiple locations, the protein was considered extracellular as long as one annotation was “extracellular space”.

Protein stability was estimated by calculating the Instability Index (II) based on its primary sequence [12]. The instability index for a given protein was calculated by the summation of the dipeptide instability weight values (DIWV) and then normalized to the length of the sequence. As recommended by Guruprasad et al. [12] , proteins with values less than 40 were defined as stable, whereas those with values greater than 40 were annotated as unstable.

### Statistical analysis

We used logistic regression to estimate or predict the probability that a protein observed in tissue will be observed in plasma. The equation relates the log odds ratio to one or more predictors. Specifically, variations estimated were spectral count (reflective of protein abundance), cellular location (whether it is extracellular), protein stability, and number of theoretical tryptic peptides (reflective of the protein length). We also tested an interaction term among cellular location, the stability score and spectral count, but those terms were not statistically significant and so were removed. The final equation is given by:
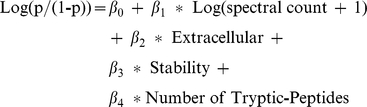



The value β_0_ represents the baseline probability and is generally not of interest given the relatively large tumor burden in the mouse model used here compared to humans. Other coefficients represent the increase (decrease) in log-odds of being detected based on the value; for practical purposes one can interpret exponential of these values as the increase in probability for the low probability events.

## Supporting Information

Figure S1 Density plots of protein instability index scores of tumor proteins detected or not in plasma. X axis is the protein instability index scores and y axis is density. The green line is the distribution of tumor proteins observed in plasma and black one is tumor proteins not detected in plasma. Proteins with instability score of <40 are stable proteins as suggested by Guruprasad et al. [12].
(TIF)

Table S1 Proteins identified in the tumor and plasma of the A431 human cell lines xenografted mouse model.
(XLS)
