# Supplementary figures and images for: Impact of Protein Stability, Cellular Localization, and Abundance on Proteomic Detection of Tumor-Derived Proteins in Plasma
Source: PLoS One. 2011 Jul 29;6(7):e23090. doi: 10.1371/journal.pone.0023090 (PMC3146523; doi:10.1371/journal.pone.0023090)

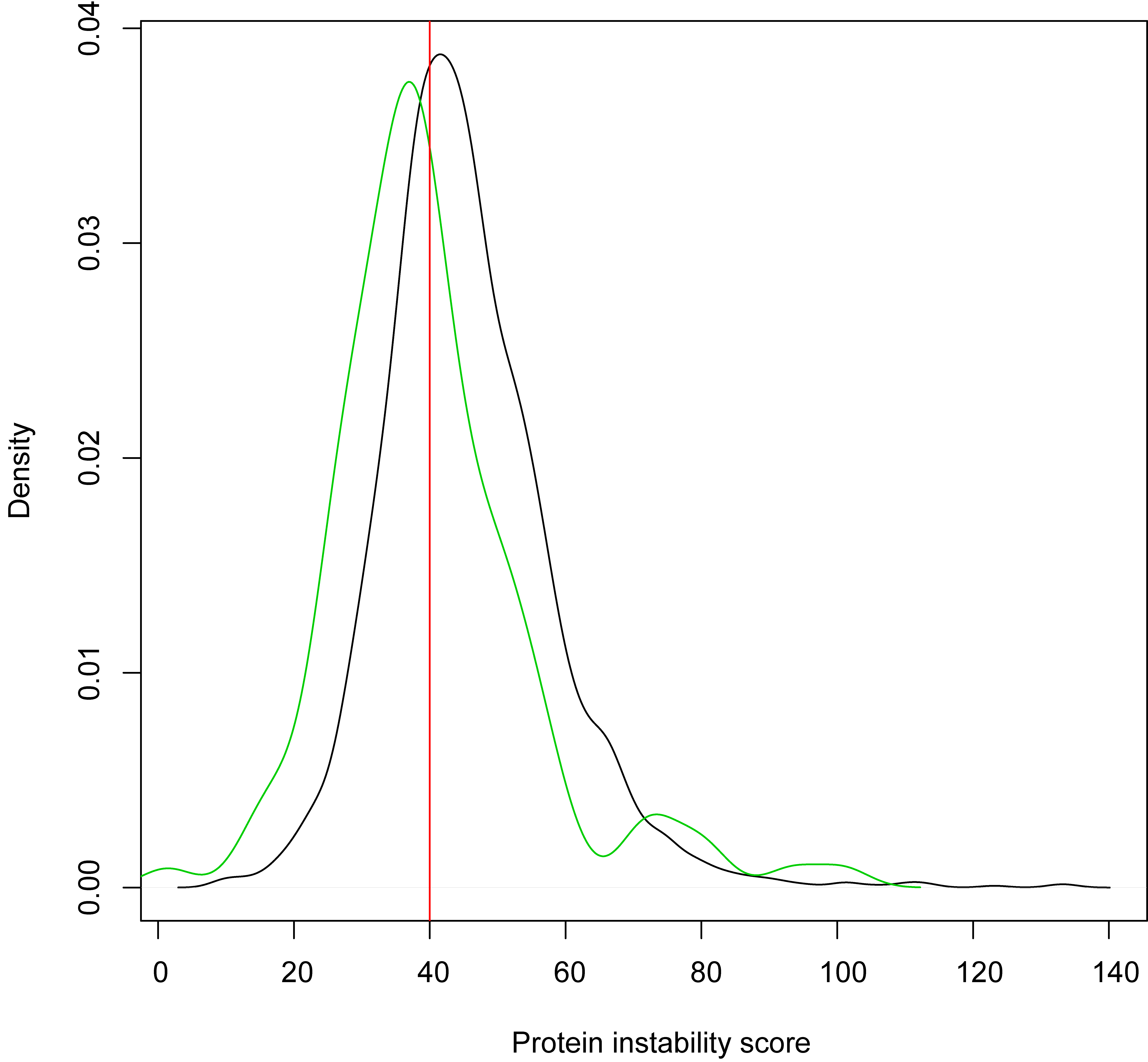

Supplement: Figure S1 — Density plots of protein instability index scores of tumor proteins detected or not in plasma. X axis is the protein instability index scores and y axis is density. The green line is the distribution of tumor proteins observed in plasma and black one is tumor proteins not detected in plasma. Proteins with instability score of <40 are stable proteins as suggested by Guruprasad et al. [12]. (TIF) [file pone.0023090.s001.tif]
